# What kind of illness is anorexia nervosa? Revisited: some preliminary thoughts to finding a cure

**DOI:** 10.1186/s40337-023-00944-3

**Published:** 2023-12-11

**Authors:** S. Touyz, E. Bryant, K. M. Dann, J. Polivy, D. Le Grange, P. Hay, H. Lacey, P. Aouad, S. Barakat, J. Miskovic-Wheatley, K. Griffiths, B. Carroll, S. Calvert, S. Maguire

**Affiliations:** 1 InsideOut Institute for Eating Disorders, Faculty of Medicine and Health, The University of Sydney and Sydney Local Health District, Sydney, Australia; 2 Department of Psychology, University of Toronto, Toronto, Canada; 3 Department of Psychiatry and Behavioral Sciences, University of California, Parnassus Heights, San Francisco, USA; 4 Translational Health Research Institute, School of Medicine, Western Sydney University, Campbelltown, Australia; 5 Mental Health Services SWSLHD, Campbelltown, Australia; 6 Population Health Research Institute, St George’s, University of London, London, UK; 7 Australian Eating Disorder Research and Translation Centre, The University of Sydney, Sydney, Australia; 8 Healing Conversations, Perth, WA Australia

## Abstract

Two decades have elapsed since our publication of ‘What kind of illness is anorexia nervosa?’. The question remains whether our understanding of anorexia nervosa and its treatment thereof has evolved over this time. The verdict is disappointing at best. Our current gold standard treatments remain over-valued and clinical outcomes are modest at best. Those in our field are haunted by the constant reminder that anorexia nervosa carries the highest mortality rate of any psychiatric disorder. This cannot continue and demands immediate action. In this essay, we tackle the myths that bedevil our field and explore a deeper phenotyping of anorexia nervosa. We argue that we can no longer declare agnostic views of the disorder or conceive treatments that are “brainless”: it is incumbent upon us to challenge the prevailing zeitgeist and reconceptualise anorexia nervosa. Here we provide a roadmap for the future.

In our essay over two decades ago, we described anorexia nervosa as follows:

“*Anorexia nervosa is a mental and physical disease that was recognised in France in the 19*th* century, usurped for England by Queen Victoria’s physician, and subsequently adopted by many thousands of Americans. According to the prevailing grand narratives embodied in DSM-IV and ICD 10, it is merely a part of the spectrum of eating disorders. This categorisation not only distorts our view of the illness, but also trivialises its seriousness (Beumont and Touyz* [1]*)*.”

It is not difficult to be disillusioned with our current concepts of anorexia nervosa (AN). Little has changed over the past 20 years since the publication of “*What kind of illness is anorexia nervosa?*”[1]. One incontrovertible fact about AN remains—it takes time to recover [2]. Despite decades of research into psychological interventions, and to a lesser extent pharmacotherapy, AN continues to have the sad distinction of having the highest mortality rate of all of the psychiatric disorders [3]. Researchers working in illnesses such as diabetes can point to their innovation in developing GLP1 agonists [4], and those in surgery to the advances in key-hole interventions [5] to manage, and in many cases even cure, people of their condition. Contrastingly, researchers and clinicians in the field of AN must accept that only 30 percent of those who survive the illness at ten years are fully recovered [6]. Schmidt and Campbell lamented that “*AN in adulthood remains markedly persistent and difficult to treat, with the holy grail of an effective, replicable outpatient treatment remaining highly elusive*” [7, 8]. It is fair to say this picture is somewhat more optimistic for patients who are rapidly treated in adolescence, providing this treatment is in the outpatient domain where families are actively engaged in support of the young person’s recovery. Moreover, such family-based approaches can significantly reduce the need for inpatient treatment, the latter often associated with high rates of relapse and readmission to inpatient settings [9, 10]. A precise explication for why treatment outcome in adolescence might be more favourable than in adulthood is complex. One hypothesis to consider here is that AN presenting in early adolescence is a ‘different’ syndrome than when presenting in adulthood in that the relational processes in adolescent AN are less compromised, which in turn makes recovery more likely [11]. Schmidt and Campbell [6, 7] also drew attention to the lack of innovation in psychosocial treatments to date and felt it to be unlikely that any future breakthroughs in treating AN would emanate out of the talking therapies alone [8]. Kaye and colleagues (2015) have rubbed further salt into the wound by declaring that the field has fallen behind other psychiatric disorders in terms of the understanding of responsible brain circuitry and pathophysiology and agree that the treatment of AN can no longer remain “brainless” [8, 12]. Bulik has further exposed this unpalatable truth by declaring that we have ‘…. not been paddling as hard as we can’ [13]. She later went on to say that the science of eating disorders has been held back by decades of “misunderstanding and misconceptions” and that there has been an ongoing promulgation of myths pertaining not only to the aetiology of AN, but as to the clinical effectiveness of treatment, as well as the prospects for recovery/cure [14].

There has been a proliferation of eating disorders in succeeding editions of the American Psychiatric Association’s Diagnostic and Statistical Manual of Mental Disorders (DSM). It has grown from one diagnostic group to eight, and each warrants our attention [15], all contributing significantly to the burden of disease in eating disorders. But the profound suffering of those with severe and enduring anorexia nervosa (SE-AN) as well as the heavy financial load that it places on their carers continues unabated [16].

It is perhaps tempting to focus our energy on repairing over-burdened mental healthcare systems post COVID-19; however, the more vexing challenge is perhaps addressing the deeper malady at play which lies at the root of the problem: what exactly is AN? Is it a misperception of body image as Bruch alluded to [17], or a psychotic illness? [18]. Is it a phobia of normal body weight as Crisp described? [19]. Garner and Bemis [20] drew similarities between the severe psychological and physiological symptoms of malnutrition observed in the Minnesota Starvation Study [21] with those of the emaciated patient with AN, yet refeeding to the expected body weight rarely in itself guarantees full recovery from the illness [22]. Is it a learned habit as proposed by Strober, Walsh and Steinberg, and are such habits prone to consolidation during episodes of under-nutrition? [7, 23]. The origins of this concept can be traced back to Montaigne who summarised this phenomenon better than most:

*For in truth habit is a violent and treacherous schoolmistress. She establishes in us, little by little, stealthily, the foothold of her authority; but having by this mild and humble beginning settled and planted it with the help of time, she soon uncovers to us a furious and tyrannical face against which we no longer have the liberty of even raising our eyes (Montaigne (1580), in Graybiel* [24]*)*.

If neurobiological mechanisms are the root cause, will newer interventions such as temperament-focussed treatments be the answer? [12]. Are there data to refute some of these early propositions which might at least open the door ever-so-slightly for a reconceptualisation of AN? [25]. The room for growing our understanding of AN is boundless, but a shift in thinking is needed.

As early as 1985, Touyz et al. showed that those with AN did not misperceive or inaccurately distort their body image, but their persistent distress regarding their body shape was an affective over-evaluation rather than a factual distortion or visual misperception [26]. Yet this over-valuation of shape and weight has become the hallmark of AN and “such enduring wrong assumptions” are now being challenged [27]. Those with lived experience have been equal in their disdain regarding the social contagion of “body image” being at the core of this devastating illness. Such simplification of the complexity of AN becomes minimising, ultimately breeds stigma and misdirects the focus of research and clinical advancement. Bryant (2021), in her Lancet Psychiatry essay, has summarised this well when she asserts that “anorexia is an illness that blurs culture and pathology, and modern medicine still does not understand it” and that “the idea that something this powerful is merely a gesture of vanity is not only laughable, it is insulting” [28].

It is important to remember here that the earliest descriptions of AN by Moreton [22], Gull [29], and Lasegue [30] did not refer to any concerns about shape or weight, and it would therefore seem that that such a depiction of this illness is perhaps a more recent development in the history of AN [31]. Consequently, it has led to speculation that there could in fact be fat or weight phobic and non-fat phobic cases of AN [32, 33]. Moreover, in a recent published study by our group, 5 of 21 AN patients admitted to hospital had beliefs of delusional intensity rather than merely having an over-valued idea [34]. To these arguments, one must add the important contribution of the Anorexia Nervosa Genetics Initiative (ANGI) study [35], which delineated many of what might be considered core symptoms of AN, but also came to the conclusion, albeit preliminary, that it was not only a psychiatric disorder but a metabolic one as well [36].

Is weight recovery from AN equivalent to actual recovery? Our group contrasted those who had gained significant weight (and can be considered weight recovered) to those who had in fact more fully recovered on several measures (including Eating Disorders Examination score and psychosocial adjustment) [37]. There were subtle differences between these two groups, suggesting the deeper more damaging psychological roots characteristic of AN had not diminished despite significant weight gain. There is an abundance of clinical confirmation as to the veracity of this contention, in that patients often allude to profound and life-disrupting distress caused by their persistent illness whilst at exactly the same time, their caregivers and families report their relief at the weight gain [38]. Caregivers and families, together with the therapeutic team appear unable to understand the overwhelming anxiety and phobic distress at the core of their loved one’s suffering has either remained constant, or at times even become exacerbated despite significant gains in weight. In many cases, they go on to lose weight again to alleviate and communicate that distress [38, 39]. This is not to negate that weight gain is essential for recovery in the emaciated patient with AN, but unfortunately for many, weight recovery alone is not the silver lining as the “monster within” continues unabated.

We have also referred to a biological candidate marker for AN. In her ground-breaking ERP studies, Hatch et al. [40] was able to show that emotionally elicited ERPs pertaining to facial expression did not change throughout weight gain and remained depressed relative to controls. In not too dissimilar a vein, the over-active Default Mode Network (DMN) is now being targeted in innovative studies using psilocybin assisted psychotherapy [41]. Koning and Brietzke [41] in their narrative review on the potential role of Psilocybin Assisted Psychotherapy (PAP) in eating disorders, describe a 1959 French clinical case study of a patient with treatment resistant AN who received two doses of psilocybin. This resulted in her gaining insights into the root causes of her disorder with an almost immediate improvement in mood, and longer-term weight gain. They go on to provide a cogent argument that a disturbed neurotransmitter signalling may lie at the heart of the aetiology of this disorder. There is now emerging evidence to implicate both neurostructural changes in AN as well as abnormalities in reward and somatosensory processing networks [42, 43]. Koning and Brietzke postulate that PAP may target many of the core aspects of AN including (a) serotonergic function, (b) abnormal eating behaviours, (c) depressive symptoms, (d) cognitive flexibility, (e) anxiety, (f) distress and avoidance of feared foods, as well as (g) acceptance of weight gain [41]. Much enthusiasm abounds with regards to psychedelics in the treatment of psychiatric disorders [44], however the clinical efficacy of these treatments remains to be determined and it is unlikely to be a panacea for “all that ails”.

The outcome data of such studies are eagerly awaited as they may identify critical differences in brain function in AN. As Kaplan points out “AN hijacks the neuronal system of the brain and pathologises it” (personal communication). Williams has started to map the brain circuitry in depression which could ultimately change the clinical landscape for that illness. For example, she refers to a large biomarker prediction study that indicates amygdala hyperactivation consistent with a negative bias biotype might help to delineate those patients who are less likely to respond to alternative types of antidepressants such as a dual-action serotonin–noradrenaline reuptake inhibitor [45]. Could the brain circuitry of AN be mapped in a similar way to that of depression? Both genetic and clinical research point to an almost hatred /disavowal of self, neophobia, unimaginable anxiety, intrapersonal and interpersonal distress, perseveration and rigidity, fear of failure, maturity fears, a disconnect between perception of illness severity and even impending death and a feeling of not deserving to eat, which are stoically defended despite evidence to the contrary [2]. As stated previously, at times these reach delusional intensity.

Our current treatments either focus disproportionally on overvaluation of shape and weight or on refeeding [22, 46, 47]. We accept both are essential for ultimate recovery. There are many clinical examples of AN patients who attain a normal weight who show a rapid and somewhat remarkable recovery after re-engaging with life as it was prior to weight loss. However, others struggle, despite having received evidence-based interventions and go on to progress to a severe and often enduring form of this disorder. What separates out the 30 percent who recover within 10 years from the 30 percent who require 10–20 years to achieve the exact same outcome, and the 40 percent who appear to never recover or die an often-tragic early death after years of suffering, for both patient and their carers? [6]. It does not seem implausible that the lengthy delay to full recovery after more than a decade of illness may indicate the brain healing itself, but only if an optimum weight is maintained whilst at the same time challenging the debilitating symptoms of the disorder. Such brain plasticity is now well known [48]. The time may have come to embark on a harm minimisation strategy [49] using universally accepted rehabilitation concepts (e.g., supported accommodation, recreational and vocational opportunities) as has been established for schizophrenia [50]. It may well be that those with SE-AN who appear to show a remarkable recovery after years of illness are in fact those who were able to realign their disrupted neuronal system by maintaining a more optimum weight and at the same time dealing with the core psychopathological characteristics of the disorder.

With recent developments in the study of genetics and epigenetics, metabolism, neural networks and personalised medicine [51], we are at the cusp of a paradigm shift which augers well for the future. For more than a century we have created a straw house, just like the three little pigs in the well-known children’s fable. This has served its purpose until now as clinicians have an imperative to treat and individuals with AN deserve nothing less than to be offered, at least initially, an evidence-based treatment. But these treatments continue to have modest recovery rates [6] and are wearing thin despite attempts to boost their clinical efficacy. How long will it take for the big bad wolf to come along and ‘huff-and-puff and blow the house down’? Only time will tell!

What will the future look like in the brave new world of eating disorders and how will we get there? The world recently witnessed the break-neck speed with which research related to COVID-19 was translated into practice [5]. With the will and appropriate funding, change can happen swiftly. The eating disorders field needs to build on the scientific foundations laid, and in many countries there are now ground-breaking research initiatives such as government-funded centres for excellence to enable this. This will ensure a sophisticated research infrastructure and workforce at the ready for the next exciting chapter in our understanding of what eating disorders are and how they can be best treated [52, 53].

When it comes to the oldest eating disorder in the DSM [15], anorexia nervosa, it is “groundhog day”. We need to start again. However painful this may be, it must surely be done. This is not to throw the proverbial baby out with the bathwater – much has been achieved over the past decades including important theory developments and research demonstrating the safety of rapid refeeding without the over-arching nemesis of refeeding syndrome [54], or undertaking refeeding in the home environment [55]. But the well-worn assumption that AN is essentially a phobia about body image and that refeeding to a healthy weight constitutes full recovery should be reconsidered. AN is a complex psychiatric/metabolic disorder with roots firmly entrenched in early childhood characterised by a heightened degree of anxiety, lack of reward sensitivity, the avoidance of novelty seeking and a fragile self-esteem with a desperate need for sameness [56–59]. A marked fear of failure, and early indications of reluctance to engage in interpersonal relationships is at times confused by the suggestion that AN is somehow implicated in Autism Spectrum Disorder (ASD)—although this is not to say that some with AN may in fact have a dual diagnosis in this regard [60].

Although the Eating Disorder Examination (EDE) [61] has become the “gold standard” in the assessment of eating disorders and has enabled a high level of comparison between published studies, it has an unfortunate bias in that it conceptualises AN as a disorder with overvaluation of weight and shape at its core. The time has arrived to better delineate the phenomenology of AN [58], and then construct targeted treatments in the true spirit of precision psychiatry. To do so, a new multiaxial assessment instrument is needed that provides a comprehensive profile of each and every patient diagnosed with AN so that the complexities inherent in each case can be better evaluated and then targeted in intervention [62].

What would such a multiaxial assessment look like? What is needed is a scale that includes a comprehensive psychological profile of the core characteristics of AN, thus eliminating the need for the commonly used psychometric instruments such as the DASS, Beck Depression or Anxiety inventories, WSAS [63–66], self -esteem measures, perfectionism inventories, quality of life measures, to name just a few. We began this work developing the first co-designed (before the concept was fashioned) deep multi-axial assessment purely designed to measure the core psychological features of anorexia nervosa and assess their manifestation along severity axes providing a deeper understanding of the phenotype (the CASIAN; [67, 68]. This work needs to be extended and broadened to include other parameters, although the second axis could measure the stage of illness as the illness changes with regards to severity over time [69]. The third axis may include the laboratory investigations routinely administered such as bloods, biochemistry, liver function, ECG and bone densitometry scans. A fourth axis could include brain circuitry based upon EEG and fMRI analyses. A biological candidate marker for AN already exists [40], and as this rapidly evolving phase of discovery gains momentum, this axis will come into its own. A further axis should provide a comprehensive neuropsychological profile based upon well-established research criteria developed by Tchanturia et al. and others [70, 71]. Lastly, an additional axis devoted to functional outcomes is warranted. Improvements in everyday functioning are meaningful treatment outcomes for patients [72], and despite current illness, AN patients have enduring functional strengths which could be integrated into treatment [73]. What is so remarkable about AN is the oft-observed degree of resilience when it comes to academic achievement, or the ability to outperform others in a scholastic or work environment. This phenomenon has been noted in the genetic studies undertaken thus far [36], and although perfectionistic and maladaptive because of its extremes, does offer a strength to be better utilised in therapy.

Finally, the subtyping of restrictive versus binge/purge AN, which has been integral to each and every DSM iteration, may have reached its used-by-date. Although somewhat diligently recorded in almost every published study, and if not provided then without doubt would be requested during the review process, this distinction adds little to overall clinical care. Pierre Beumont was not only one of the founding fathers in the field of eating disorders, but a visionary scientist as well. While he was the first to point to the heterogeneity of AN and the need to subtype the disorder [74], a new subtyping is proposed here similar to an early model of depression [75]. A novel subtyping of reactive versus endogenous AN needs scientific exploration (Polivy, 2023 personal communication). It is proposed that in those patients with AN, where a clear precipitant is able to be identified (Reactive AN), existing evidenced-based treatments may be clinically effective; but, perhaps less so for those who have a more endogenous onset without an obvious precipitating factor or event (Endogenous AN). Patients with more complex presentations also appear less likely to respond to talk therapies, and may need a different, possibly more biological, approach. It is the latter group to whom the term “treatment resistant” is likely to be applied, with blame often attributed to the person with the illness. It is, however, the therapist who might be inadequate here, as the existing (psychological) treatments may not have the clinical power to effect change in this seriously ill and distressed cohort.

We cannot deny the indisputable fact that patients with AN continue to suffer (many for years and even decades) and die from this serious disorder. Patients in continental Europe have been approved for euthanasia and reports on “palliative care” are increasing. The desperate need to avoid further suffering is often openly expressed by those with a lived experience and their carers alike. However, others have expressed caution— asserting that in the absence of a clinically effective evidence-based treatment, it is unwise to talk about someone being treatment resistant, and further, that many patients with AN either receive no treatment at all − or at best inadequate treatment. Are these the advances we desperately wanted in our field in 2023? We suspect not. The time for complacency has ended and the need to find a cure for AN has arrived. We have brilliant minds at work, but rather than working in silos we need to work together with colleagues not just from inside our field, but also from outside our immediate field to finally put the conundrum of AN to rest. Bulik has likened the field of eating disorders to an island, suggesting we have not been “gregarious enough in engaging external scientists in our work” [14]. We also need to find ways to overcome the issue of under-funding in eating disorder research that contribute to the maintenance of the status quo [76, 77]. It is now almost a century and a half since Gull [29] described AN, and those enduring the illness and their carers cannot wait any longer. We owe it to them.

So, what might the treatment of AN look like in a decade’s time? Kan and colleagues have given us a glimpse into the futuristic world of AN treatment and a possible roadmap to get there [51]. They provide a cogent argument that the time has now arrived to focus our research initiatives on developing new interventions that reduce the translational gap between emerging findings in neuroscience and the clinic. The days of agnostic assumptions in this regard are numbered.

Thus, we may need to tailor treatment and supplement intervention by targeting specific elements of risk and resilience. This would require a deeper phenotyping to examine facets of the core psychopathology including social and interpersonal function, reward reinforcement, anxiety sensitivity, cognitive styles and other biomarkers [51].

We encourage others to build upon this model or to provide alternative ones. Fernandez-Aranda and colleagues have already reminded us that “necessity is the mother of invention” and the aftermath of COVID 19 will no doubt lead to changes in our therapeutic models with the introduction of “more efficient and effective mixed methods of connection and a more personalized treatment palette as to what and how might work best for whom” [78].

So, what might the smorgasbord of innovative treatment modalities look like in the AN clinic of the future? Both Treasure et al. and Stengel and Giel have already begun to explore “emerging therapeutic targets” that could provide the armamentarium for treatment delivery in the next decade [79, 80]. These will not only include the eating disorder phenotype such as cognitive, social and emotional difficulties, but also compounds other than olanzapine and antidepressants. The list grows longer day by day with further interest in lithium, ketamine, psilocybin, opioids, endocannabinoids as well as hormones such as oestrogen, histamine, oxytocin, leptin, growth hormone, ghrelin and nesfatin-1 [81]. These could be supported by innovations that better target eating behaviour habits and underlying processes that focus particular attention on implementation interventions, exposure-based therapies, inhibition training as well as disruptions to food cravings. Furthermore, neuromodulatory treatments that include non-invasive brain stimulation (NIBS) and deep brain stimulation (DBS) are being actively explored, however, caution should be exercised before we rush into interventions based on preliminary hypotheses or limited evidence. Further systematic research will be needed to determine the ultimate clinical success of these novel treatments, which must also be balanced by the risk of doing harm and making sure to adhere to the first rule in medicine “primum non nocere”.

Co-design is the new mantra of the day but rarely is it implemented in the manner that it was advocated and unfortunately tokenism abounds. Stengel and Giel so aptly point out that if our desired aim is to increase the acceptability and eventual adoption of novel therapeutics then “… it will be an important next step to increase integration of lived experience by patients and carers into the whole clinical research process” [80]. The status quo can no longer prevail. Science does not take kindly to attempts to change the existing zeitgeist but there now appears to be an unstoppable groundswell and determination to do exactly that in both our understanding and delivery of efficacious treatment(s) in AN. Existing clinical guidelines will become increasingly challenged and much careful thought and deliberation will need to be given to future iterations of DSM and ICD as the avalanche of new and exciting research findings come into play.

It is not expected that those who read this commentary will agree with all the propositions enunciated above, but it is hoped that it may spur others to action as it is likely to be a collective enterprise that ultimately bears the fruit of success. [16]. Opportunities for increasing research spending and providing opportunities for cross-collaborative research will go some way to enhancing translational research in the eating disorder field. However, any such enterprise must embrace the views of those with lived experience and their carers. They know better than most as to what this “monster within” does to often brilliant minds. We should refrain from our well-worn mantra of improving clinical effectiveness to the much loftier aspiration of finding a cure for AN. It is now within our grasp and time is of the essence. This journey has already commenced and the quotation from Noam Chomsky below should provide further impetus to realise this lofty aim.

“Optimism is a strategy for making a better future. Because unless you believe that the future can be better, it's unlikely you will step up and take responsibility for making it so. If you assume that there's no hope, you guarantee that there will be no hope.”—Noam Chomsky

## Data Availability

Not applicable.

## References

[1] Beumont PJV, Touyz SW (2003). What kind of illness is anorexia nervosa?. Eur Child Adolesc Psychiatry.

[2] Touyz S, Strober M (2016). Managing the patient with severe and enduring anorexia nervosa.

[3] Arcelus J, Mitchell AJ, Wales J, Nielsen S (2011). Mortality rates in patients with anorexia nervosa and other eating disorders: a meta-analysis of 36 studies. Arch Gen Psychiatry.

[4] Baynes KC (2010). The evolving world of GLP-1 agonist therapies for type 2 diabetes. Ther Adv Endocrinol Metab.

[5] Touyz S, Lacey H, Hay P (2020). Eating disorders in the time of COVID-19. J Eat Disord.

[6] Eddy KT, Tabri N, Thomas JJ, Murray HB, Keshaviah A, Hastings E (2017). Recovery from anorexia nervosa and bulimia nervosa at 22-year follow-up. J Clin Psychiatry.

[7] Walsh BT (2013). The enigmatic persistence of anorexia nervosa. Am J Psychiatry.

[8] Schmidt U, Campbell IC (2013). Treatment of eating disorders can not remain ‘brainless’: the case for brain-directed treatments. Eur Eat Disord Rev.

[9] Eisler I, Simic M, Fonagy P, Bryant-Waugh R (2022). Implementing service transformation for children and adolescents with eating disorders across England: the theory, politics, and pragmatics of large-scale service reform. J Eat Disord.

[10] Bentz M, Pedersen SH, Moslet U (2021). An evaluation of family-based treatment for restrictive-type eating disorders, delivered as standard care in a public mental health service. J Eat Disord.

[11] le Grange D, Eisler I (2009). Family interventions in adolescent anorexia nervosa. Child Adolesc Psychiatr Clin N Am.

[12] Kaye WH, Wierenga CE, Knatz S, Liang J, Boutelle K, Hill L (2015). Temperament-based treatment for anorexia nervosa. Eur Eat Disord Rev.

[13] Bulik CM (2013). Are we really paddling as fast as we can? reflections on why eating disorders treatment and research always seem to be one step behind: commentary on hay, mitchell, and stice & becker: prevention and treatment. Int J Eat Disord.

[14] Bulik CM (2016). Towards a science of eating disorders: Replacing myths with realities: the fourth Birgit Olsson lecture. Nord J Psychiatry.

[15] American psychiatric association. Feeding and eating disorders. Diagnostic and statistical manual of mental disorders. 5th Ed, Text Revision 2022.

[16] Pépin G, King R (2013). Collaborative care skills training workshops: helping carers cope with eating disorders from the UK to Australia. Soc Psychiatry Psychiatr Epidemiol.

[17] Bruch H (1962). Perceptual and conceptual disturbances in anorexia nervosa. Psychosom Med.

[18] Rodgers E, Marwaha S, Humpston C (2022). Co-occurring psychotic and eating disorders in England: findings from the 2014 adult psychiatric morbidity survey. J Eat Disord.

[19] Crisp AH (1970). Premorbid factors in adult disorders of weight, with particular reference to primary anorexia nervosa (weight phobia). A literature review. J Psychosom Res.

[20] Garner DM, Bemis KM (1982). A cognitive-behavioral approach to anorexia nervosa. Cogn Ther Res.

[21] Keys A, Brožek J, Henschel A, Mickelsen O, Taylor HL. The biology of human starvation. (2 vols). 1950.

[22] Phillipou A, Rossell SL, Castle DJ (2018). Anorexia nervosa or starvation?. Eur J Neurosci.

[23] Steinglass J, Walsh BT (2006). Habit learning and anorexia nervosa: a cognitive neuroscience hypothesis. Int J Eat Disord.

[24] Graybiel AM (2008). Habits, rituals, and the evaluative brain. Annu Rev Neurosci.

[25] Touyz S, Hay P (2015). Severe and enduring anorexia nervosa (SE-AN): in search of a new paradigm. J Eat Disord.

[26] Touyz SW, Beumont PJV, Collins JK, Cowie I (1985). Body shape perception in bulimia and anorexia nervosa. Int J Eat Disord.

[27] Gutiérrez E, Carrera O (2021). Severe and Enduring anorexia nervosa: enduring wrong assumptions?. Front Psychiatry.

[28] Bryant E (2021). Anorexia: the great taboo. Lancet Psychiatry.

[29] Gull WW (1874). Anorexia nervosa (apepsia hysterica. anorexia hysterica). Trans Clin Soc.

[30] Lasègue E-C (1873). De l'anorexie hystérique Arch gén méd.

[31] Russell GF (1970). Anorexia nervosa: its identity as an illness and its treatment. Modern Trends Psychol Med.

[32] Lee S, Ho TP, Hsu LG (1993). Fat phobic and non-fat phobic anorexia nervosa: a comparative study of 70 Chinese patients in Hong Kong. Psychol Med.

[33] Korn J, Vocks S, Rollins LH, Thomas JJ, Hartmann AS (2020) Fat-Phobic and Non-Fat-Phobic Anorexia Nervosa: A Conjoint Analysis on the Importance of Shape and Weight. Front Psychol 11: 90. 10.3389/fpsyg.2020.00090 PMC7005216 32082227

[34] Barton R, Aouad P, Hay P, Buckett G, Russell J, Sheridan M (2022). Distinguishing delusional beliefs from overvalued ideas in anorexia nervosa: an exploratory pilot study. J Eat Disord.

[35] Thornton LM, Munn-Chernoff MA, Baker JH, Juréus A, Parker R, Henders AK (2018). The anorexia nervosa genetics initiative (ANGI): overview and methods. Contemp Clin Trials.

[36] Duncan L, Yilmaz Z, Gaspar H, Walters R, Goldstein J, Anttila V, Bulik-Sullivan B, Ripke S (2017). Eating disorders working group of the psychiatric genomics consortium, Thornton L, Hinney. A significant locus and metabolic genetic correlations revealed in genome-wide association study of anorexia nervosa. Am J Psychiatry.

[37] Windauer U, Beumont PJV, Lennerts W, Talbot P, Touyz SW (1993). How well are ‘cured’ anorexia nervosa patients?: an investigation of 16 weight-recovered anorexic patients. Br J Psychiatry.

[38] Barko EB, Moorman SM (2023). Weighing in: qualitative explorations of weight restoration as recovery in anorexia nervosa. J Eat Disord.

[39] Eiring K, Wiig Hage T, Reas DL (2021). Exploring the experience of being viewed as “not sick enough”: a qualitative study of women recovered from anorexia nervosa or atypical anorexia nervosa. J Eat Disord.

[40] Hatch A, Madden S, Kohn MR, Clarke S, Touyz S, Gordon E (2010). Emotion brain alterations in anorexia nervosa: a candidate biological marker and implications for treatment. J Psychiatry Neurosci.

[41] Koning E, Brietzke E. Psilocybin-assisted psychotherapy as a potential treatment for eating disorders: a narrative review of preliminary evidence. Trends Psychiatry Psychother. 2023. May 1 (Ahead Of Print). 10.47626/2237-6089-2022-0597 PMC11790131 37126863

[42] Su T, Gong J, Tang G, Qiu S, Chen P, Chen G (2021). Structural and functional brain alterations in anorexia nervosa: a multimodal meta-analysis of neuroimaging studies. Hum Brain Mapp.

[43] Russell H, Aouad P, Le A, Marks P, Maloney D, Touyz S, Maguire S (2023). Psychotherapies for eating disorders: findings from a rapid review. J Eat Disord.

[44] Peck SK, Shao S, Gruen T, Yang K, Babakanian A, Trim J (2023). Psilocybin therapy for females with anorexia nervosa: a phase 1, open-label feasibility study. Nat Med.

[45] Williams LM (2016). Precision psychiatry: a neural circuit taxonomy for depression and anxiety. Lancet Psychiatry.

[46] Phillipou A, Castle DJ, Rossell SL (2018). Anorexia nervosa: eating disorder or body image disorder?. Aust N Z J Psychiatry.

[47] Glashouwer KA, van der Veer RML, Adipatria F, de Jong PJ, Vocks S (2019). The role of body image disturbance in the onset, maintenance, and relapse of anorexia nervosa: a systematic review. Clin Psychol Rev.

[48] Doidge N. The brain that changes itself: stories of personal triumph from the frontiers of brain science: Penguin; 2007.

[49] Dawson L, Rhodes P, Touyz S (2014). The recovery model and anorexia nervosa. Aust N Z J Psychiatry.

[50] Paul R. Severe and Enduring Eating Disorders: Concepts and Management. In: Hubertus H, Ignacio Jáuregui L, editors. Anorexia and bulimia nervosa. Rijeka: IntechOpen; 2019. p. Ch. 10.

[51] Kan C, Cardi V, Stahl D, Treasure J (2019). Precision psychiatry—what it means for eating disorders?. Eur Eat Disord Rev.

[52] Wonderlich S. Plenary: Developments in the training of the next generation of clinical researchers. Eat Disord Res Conf (EDRS). 2022.

[53] InsideOut institute for eating disorders. Australian eating disorders research and translation strategy 2021–2031. Sydney. 2021.

[54] Kohn MR, Madden S, Clarke SD (2011). Refeeding in anorexia nervosa: increased safety and efficiency through understanding the pathophysiology of protein calorie malnutrition. Curr Opin Pediatr.

[55] Hambleton A, Aouad P, Miskovic-Wheatley J, Le Grange D, Touyz S, Maguire S (2022). The efficacy of family treatments for adolescent anorexia nervosa in specialist versus non-specialist settings: protocol for a systematic review and meta-analysis. J Eat Disord.

[56] Bulik CM, Flatt R, Abbaspour A, Carroll I (2019). Reconceptualizing anorexia nervosa. Psychiatry Clin Neurosci.

[57] Zipfel S, Giel KE, Bulik CM, Hay P, Schmidt U (2015). Anorexia nervosa: aetiology, assessment, and treatment. Lancet Psychiatry.

[58] Bryant E, Aouad P, Hambleton A, Touyz S, Maguire S. ‘In an otherwise limitless world, I was sure of my limit.’† experiencing anorexia nervosa: a phenomenological metasynthesis. Front Psychiatry 2022; 13. 10.3389/fpsyt.2022.894178 PMC9376373 35978851

[59] Wierenga CE, Ely A, Bischoff-Grethe A, Bailer UF, Simmons AN, Kaye WH (2014). Are extremes of consumption in eating disorders related to an altered balance between reward and inhibition?. Front Behav Neurosci.

[60] Westwood H, Tchanturia K (2017). Autism spectrum disorder in anorexia nervosa: an updated literature review. Curr Psychiatry Rep.

[61] Fairburn CG, Cooper Z, O’Connor M (1993). The eating disorder examination. Int J Eat Disord.

[62] Schauenburg H, Friederich H-C, Wild B, Zipfel S, Herzog W (2009). Focal psychodynamic psychotherapy of anorexia nervosa: a treatment manual. Psychotherapeut.

[63] Lovibond PF, Lovibond SH (1995). The structure of negative emotional states: Comparison of the Depression Anxiety Stress Scales (DASS) with the Beck Depression and Anxiety Inventories. Behav Res Therapy.

[64] Beck AT, Ward CH, Mendelson M, Mock J, Erbaugh J (1961). An inventory for measuring depression. Arc General Psychiatry.

[65] Beck AT, Epstein N, Brown G, Steer RA (1988). An inventory for measuring clinical anxiety: Psychometric properties. J Consult Clin Psychol.

[66] Mundt JC, Marks IM, Shear MK, Greist JM (2002). The Work and Social Adjustment Scale: a simple measure of impairment in functioning. British J Psychiatry.

[67] Maguire S, Touyz S, Surgenor L, Crosby RD, Engel SG, Lacey H (2012). The clinician administered staging instrument for anorexia nervosa: development and psychometric properties. Int J Eat Disord.

[68] Maguire S, Surgenor LJ, Le Grange D, Lacey H, Crosby RD, Engel SG (2017). Examining a staging model for anorexia nervosa: empirical exploration of a four stage model of severity. J Eat Disord.

[69] Maguire S, Le Grange D, Surgenor L, Marks P, Lacey H, Touyz S (2008). Staging anorexia nervosa: conceptualizing illness severity. Early Interv Psychiatry.

[70] Tchanturia K, Campbell IC, Morris R, Treasure J (2005). Neuropsychological studies in anorexia nervosa. Int J Eat Disord.

[71] Stedal K, Frampton I, Landrø NI, Lask B (2012). An examination of the ravello profile—a neuropsychological test battery for anorexia nervosa. Eur Eat Disord Rev.

[72] Mitchison D, Morin A, Mond J, Slewa-Younan S, Hay P (2015). The bidirectional relationship between quality of life and eating disorder symptoms: a 9-year community-based study of Australian women. PLoS ONE.

[73] Dann KM, Hay P, Touyz S (2022). Everyday flexibility and functional milestones in anorexia nervosa: survey results from a mixed community sample. Eat Weight Disord Stud Anorexia, Bulimia Obes.

[74] Beaumont P, George C, Smart D (1976). "Dieters" and "vomiters and purgers” in anorexia nervosa. Psychol Med.

[75] Lewis A (1938). States of depression. BMJ.

[76] Marzola E, Panero M, Longo P, Martini M, Fernàndez-Aranda F, Kaye WH (2022). Research in eating disorders: the misunderstanding of supposing serious mental illnesses as a niche specialty. Eat Weight Disord Stud Anorexia, Bulimia Obes.

[77] Bryant E, Koemel N, Martenstyn JA, Marks P, Hickie I, Maguire S. Mortality and mental health funding; do the dollars add up? Eating disorder research funding in Australia from 2009 to 2021: a portfolio analysis. Lancet Reg Health Western Pacific. 10.1016/j.lanwpc.2023.100786 PMC10485676 37693868

[78] Fernández-Aranda F, Casas M, Claes L, Bryan DC, Favaro A, Granero R (2020). COVID-19 and implications for eating disorders. Eur Eat Disord Rev.

[79] Treasure J, Cardi V, Leppanen J, Turton R (2015). New treatment approaches for severe and enduring eating disorders. Physiol Behav.

[80] Stengel A, Giel K. Emerging therapeutic targets for anorexia nervosa. Expert Opin Ther Targets. 2023; 1–12. 10.1080/14728222.2023.2206954 37093017

[81] Keeler JL, Treasure J, Himmerich H, Brendle M, Moore C, Robison R (2023). Case report: intramuscular ketamine or intranasal esketamine as a treatment in four patients with major depressive disorder and comorbid anorexia nervosa. Front Psychiatry.

